# Determination of TGF*β*1 protein level in human primary breast cancers and its relationship with survival

**DOI:** 10.1038/sj.bjc.6602920

**Published:** 2006-01-10

**Authors:** S Desruisseau, J Palmari, C Giusti, S Romain, P-M Martin, Y Berthois

**Affiliations:** 1 Assistance Publique-Hopitaux de Marseille, Laboratoire de Transfert en Oncologie Biologique, Faculté de Médecine Secteur Nord, 13916 Marseille Cedex 20, France; 2 INSERM EMI 0359, Laboratoire de Cancérologie Expérimentale, IFR Jean-Roche, Faculté de Médecine Secteur Nord, Bd Pierre Dramard, 13916 Marseille Cedex 20, France

**Keywords:** transforming growth factor-beta1, breast cancer, survival

## Abstract

Transforming growth factor-beta (TGF*β*)1 is thought to be implicated in breast cancer progression. However, data about the influence of TGF*β*1 on breast cancer development are conflicting. To clarify the clinical relevance of TGF*β*1, TGF*β*1 protein level has been measured by enzyme-immoassay in 193 breast tumour samples. We found that 94.3% of patients expressed TGF*β*1 with a range of 0–684 pg mg^−1^ protein. In the overall population, an increase of tumoral TGF*β*1 was observed in premenopausal patients when compared to postmenopausal subgroup (*P*=0.0006). When patients were subdivided according to nodal status, TGF*β*1 was correlated to type-1 plasminogen activator inhibitor in the node-negative subgroup (*P*=0.040). Multivariate analysis revealed that, after lymph node status (*P*=0.0002) and urokinase-type plasminogen activator (*P*=0.004), TGF*β*1 was an independent prognostic marker for DFS (*P*=0.005) in the overall population. In the node-negative population, TGF*β*1 was the prominent prognostic factor (*P*=0.010). In the same population, Kaplan–Meier curves demonstrated that high TGF*β*1 level was correlated with a shorter disease-free survival (*P*=0.020). These data suggest that the measurement of tumoral TGF*β*1 protein level, especially for node-negative patients, might help to identify a high-risk population early in tumour progression.

Transforming growth factor-beta (TGF*β*) belongs to a superfamily of secreted polypeptides, which regulate cell proliferation, differentiation, motility and apoptosis in a variety of different cell types (Roberts and Sporn, 1990). Three TGF*β* isoforms 1–3 are ubiquitously expressed and have been detected in humans and other mammals. TGF*β*1 have been associated with both normal mammary gland development and breast carcinogenesis (Wakefield *et al*, 2000). Thus, *in vivo*, TGF*β*1 appears to regulate normal ductal and alveolar development in the mammary gland (Jhappan *et al*, 1993). Moreover, TGF*β*1 probably mediates the massive cell death and restructuring that takes place during postlactational involution of the mammary gland (Strange *et al*, 1992). Besides these physiological functions, there is considerable evidence that TGF*β*1 is implicated in several aspects of breast cancer onset and progression (Wakefield *et al*, 2000). A commonly held view is that TGF*β*1 prohibits tumour cell proliferation because TGF*β*1 is a potent growth inhibitor for nearly all epithelia *in vitro* (Roberts and Sporn, 1990). Moreover, TGF*β*1 can induce apoptosis, a process associated with tumour suppression, promote replicative senescence and exhibit negative regulation of angiogenesis (Alexander and Moses, 1995; Schwarte-Waldhoff *et al*, 2000; Perlman *et al*, 2001). Consistent with a tumour-suppressor role, transgenic mouse models have bring evidence that TGF*β*1 is able to protect against mammary tumour development *in vivo*, because either the suppression of TGF*β*1 or the inactivation of the TGF*β* signalling pathway results in loss of tumour prohibition and promotes carcinogenesis (Pierce *et al*, 1995; Bottinger *et al*, 1997). On the other hand, virally transformed tumorigenic mammary epithelial cell lines as well as most of the cell lines derived from invasive human breast carcinomas are resistant to the antiproliferative effects of TGF*β*1 *in vitro* and do not respond to treatment with TGF*β*1 *in vivo*. In a number of cases, this is attributable to inhibiting mutations in either TGF*β* type I or II receptors (Chen *et al*, 1998; Gobbi *et al*, 2000) or deregulation of the downstream signalling cascade (Xie *et al*, 2002).

In addition, there is increasing evidence that after cells lose their sensitivity to TGF*β*1-mediated growth inhibition, autocrine TGF*β* signalling may promote tumorigenesis. Consistent with a pro-oncogenic role for TGF*β*1 in late-stage cancer, elevated levels of TGF*β*1 are often observed in advanced carcinomas, and have been correlated with increased tumour invasiveness and disease progression in a variety of tumours such as malignant melanoma (Moretti *et al*, 1999) and colonic (Tsushima *et al*, 1996), ovarian (Bristow *et al*, 1999), and prostatic (Shariat *et al*, 2004) cancers. This suggests that secreting higher levels of TGF*β*1 may provide an advantage to tumour cells. Pro-oncogenic effects include direct effects of TGF*β*1 on tumoral cells, such as the stimulation of invasion and motility (Oft *et al*, 1996). Tumour TGF*β*1 may also indirectly promote cancer progression by promoting tumour vascularisation (Oh *et al*, 2000) and inhibiting mechanisms of immune surveillance (Letterio and Roberts, 1998).

In breast cancer, a number of studies have been engaged to evaluate the potential prognostic value of TGF*β*1. In most of these studies, tumoral TGF*β*1 expression has been examined either by immuno-histochemical assay or by Northern blot analysis. Although several groups demonstrated that increased TGF*β*1 was associated with more aggressive tumour behavior and poorer survival (Gorsch *et al*, 1992; Dalal *et al*, 1993; Walker *et al*, 1994), other authors reported the absence of correlation between disease progression and TGF*β*1 immunostaining or mRNA level (Dublin *et al*, 1993; Ghellal *et al*, 2000). Furthermore, a few number of studies demonstrated that TGF*β*1 was related to favorable outcome for patients with breast cancers (Murray *et al*, 1993; Kesari *et al*, 1999).

To date, quantitative determination of TGF*β*1 protein level has been performed exclusively in plasma of breast cancer patients. While some studies failed to reveal any change in plasma TGF*β* value in patients with breast cancer (Wakefield *et al*, 1995; Lebrecht *et al*, 2004), other reports demonstrate that patients with more advanced tumours have higher serum levels of TGF*β*1 (Kong *et al*, 1995; Ivanovic *et al*, 2003), suggesting that serum TGF*β*1 may reflect the severity of invasive breast cancer. However, these late studies have been performed on a small cohort of patients and potential prognostic value of TGF*β*1 has not been clearly determined. To clarify the relevance of TGF*β*1 as a prognostic marker in human breast cancer, we quantified TGF*β*1 protein level in 193 breast tissue specimens. The association between TGF*β*1 and the usual histological and biological parameters previously validated was examined. The prognostic relevance of TGF*β*1 for disease-free (DFS) and overall survival (OS) was studied for all patients by Cox multivariable analysis including the traditional prognostic markers.

## MATERIALS AND METHODS

### Patient population

This study involved 193 patients diagnosed and treated in Assistance Publique of Marseille (France) between early 1987 and late 1992. These patients were previously included in a retrospective multicenter study (Romain *et al*, 2000). Patients were selected according to the following criteria: (1) primary unilateral breast tumour; (2) previously untreated, no evidence of metastatic disease or any other malignancy at the time of diagnosis; (3) T1T2, N0N1 status according to UICC criteria; (4) <75 years old; and (5) surgery as the first treatment.

The patients were 29–74 years old at diagnosis, with a median age of 57 years. In total, 37.3% of patients were premenopausal. A total of 83 patients presented a tumour size ⩽2 cm; 48.7% of patients were node-negative (N−), 29.6% presented one to three axillary invaded nodes (N+) and 21.7% had more than three invaded nodes (N++). Among the 193 tumours graded according to Scarff, Bloom and Richardson classification (SBR), 25.5% were classified grade I, 52.8% were grade II, and 21.7% were grade III. Ductal carcinomas were diagnosed in 75% of patients, and invasive lobular carcinomas in 25% of patients.

The primary treatment was tumorectomy or quadrantectomy (92%) or modified radical mastectomy (8%) with axillary dissection, followed by radiotherapy in 98% of cases. Among the 121 postmenopausal patients, 21 received no adjuvant treatment whereas 33 received hormone therapy, 33 were treated with chemotherapy, and 34 received both treatments. Among the 72 premenopausal patients, 36 received no adjuvant therapy and 36 were treated as follow: 10 with chemotherapy, 22 with hormone therapy and four patients with both treatments. The median follow-up was 94 months (range, 1–140). At the cutoff date of this study, 16 local recurrences, 42 metastasis and 28 deaths had been recorded. Tumour samples and clinical informations were obtained under Institutional Clinical Board approval.

### Preparation of tumour tissue extracts and ER and PR assays

Tumour tissues were stored in liquid nitrogen and routinely assayed for estrogen (ER) and progesterone receptors (PR) levels, according the recommendations of the European Organization for Research and Treatment of Cancer (EORTC), as previously described (Foekens *et al*, 1989). Tumour tissues were pulverised in the frozen state with a microdismembrator (Braun, Melsungen, Germany) as recommended by the EORTC. The resulting powder was suspended in buffer containing 10 mM Tris-HCl pH 7.4, 1.5 mM EDTA, 10 mM Na_2_MoO_4_, 0.5 mM DTT and 10% glycerol. The suspension was centrifuged for 60 min at 105 000 *g* at 4°C. The high-speed supernatants (cytosols) were collected and stored in liquid nitrogen. For all samples, cytosolic protein concentration was determined using BCA assay (Pierce Chemical, Rockford, IL, USA). ER and PR levels were determined by enzyme immunoassay as described previously (Foekens *et al*, 1989). To assess the between-assay variations, in each series of tests an aliquot of a pooled breast cancer cytosol sample was analyzed.

The remaining cytosols were frozen and stored in liquid nitrogen until used for the determination of thymidine kinase (TK) enzyme activity, urokinase-type plasminogen activator (uPA), type-1 plasminogen activator inhibitor (PAi-1), and TGF*β*1.

### TK, uPA and PAi-1 assays

TK enzyme activity was measured using the Prolifigen TK Radioenzymatic Assay (Sangtec Medical, Bromma, Sweden), with the modifications recommended by the EORTC Receptors and Biomarkers Study Group (Foekens *et al*, 2001).

uPA levels were measured with the Immunobind® uPA ELISA kit and PAi-1 levels by the Immunobind® PAi1 ELISA kit (American Diagnostic, Greenwich, CT, USA), according to the instructions of the manufacturer. Inactive and active forms of uPA are all recognised by the uPA ELISA kit, as is receptor-bound uPA and uPA complexed with PAi-1 and PAi-2. PAi-1 ELISA detects latent and active forms of human PAi-1 and PAi-1 complexes. The assay is insensitive to PAi-2.

### TGF*β*1 measurement

TGF*β*1 levels in breast tumour cytosols were measured by ELISA. This assay used monoclonal antibody (R&D Systems, UK) as capture antibody and biotinylated polyclonal antibody (R&D Systems, UK) as detection antibody. The assay specifically measures active TGF*β*1 forms. To measure total TGF*β*1 present in tumour samples, biologically latent TGF*β*1 was activated by acid-treatment. For this purpose, cytosols were diluted with four volumes of DPBS buffer (2.7 mM KCl, 137 mM NaCl, 1.5 mM KH_2_PO_4_, 3.2 mM Na_2_HPO_4_, 1 mM CaCl_2_, 0.5 mM MgCl_2_, pH 7.4). Samples were then incubated for 15 min at room temperature in the presence of 0.02 vol of 1 N HCl, then neutralised with equal volume of 1 N NaOH. ELISA analysis was performed in 96-well plates following the instructions of the manufacturer (R&D Systems, UK). Recombinant human TGF*β*1 (R&D Systems, UK) was used as standard at 0–1000 pg ml^−1^. A preliminary evaluation was performed to assess the buffer compatibility and the parallelism of sample dilutions. The inter- and intra-assay (*n*=10) CVs of a pool of tumour extracts with mean value of 134.1 pg TGF*β*1 per mg protein were 7.5 and 3.9% respectively.

### Statistical analysis

The strength of the associations of TGF*β*1 with other variables was tested with Spearman rank correlation. The associations of TGF*β*1 (used as continuous variables) with other variables (used as grouping variables) were examined using Mann–Whitney *U* test (two categories), or in the case of more than two ordered categories by Kruskal–Wallis test. Survival curves were generated using the method of Kaplan and Meier and the log-rank test for trend was used to examine survival data. For the univariate survival analysis, DFS time (the interval between date of surgery and primary failure defined as a locoregional and/or distant recurrence) and OS time (the interval between date of surgery and death by any cause) were used as follow-up parameters. *P*-values ⩽0.05 were considered as significant.

Cox multivariate regression analysis was used to evaluate the prognostic value of TGF*β*1 in the overall or N+ or N− populations. Multivariate analysis was performed with variables eliminated in a step-down fashion. Variables with a *P*⩽0.05 were retained in the final multivariate models. Hazard ratios (HR) derived from the estimated regression coefficients, are presented with their 95% confidence intervals (CI).

Variables were categorised as follows: age (50 or younger, and older than 50), pathological tumour size (⩽20 mm or >20 mm), menopausal status (premenopausal *vs* postmenopausal), pathological nodal status (N−, none; N+, 1 to 3; N++, more than 3), histologic grade (SBR grade I, II or III), and histologic type (ductal *vs* lobular).

In regard to the variations of ER levels observed in premenopausal *vs* postmenopausal patients (39), all tumours were considered to be estrogen receptor-negative (ER−) if ER values <15 fmol mg^−1^ protein; for the premenopausal population, tumours with ER 15–205 fmol mg^−1^ protein (75th percentile) were classified ER+, whereas tumours of postmenopausal patients were considered as ER+ when ER level was 15–377 fmol mg^−1^ protein (75th percentile). In both pre- and post-menopausal populations, ER++ represents tumours with ER values exceeding the 75th percentile. In all cases, the tumours were considered to be PR-positive if values exceeded 20 fmol mg^−1^ protein. For all others biological parameters, cutpoints corresponded to the 25th and 75th percentiles of the distribution (see Table 1).

## RESULTS

### Clinicopathological characteristics

The clinicopathological characteristics of the patients are presented in Table 1. Patients were characterised according to their age, hormonal (menopausal) and steroid receptor status, tumour grade according the SBR grading system, histology and size of the tumour, and the axillary nodal status.

### Biological characteristics of the breast cancer samples analyzed

The distribution of biological factors in breast cancer samples are listed in Table 2. A wide inter-patient variability in the levels of all the parameters measured could be observed. ER, PR and TK levels were previously determined in our laboratory and integrated elsewhere in other published study (Romain *et al*, 1995, 2000). uPA and PAi-1 levels ranged from 0.01 to 1.39 ng mg^−1^ protein (median, 0.20) and from 0.27 to 54 ng mg^−1^ protein (median, 6.0), respectively. TGF*β*1 was detectable in 94.3% of samples and its concentration ranged from 0 to 684 pg mg^−1^ protein, with a median at 86.7 pg mg^−1^ protein.

### Relationships between TGF*β*1 and clinicopathological and biological parameters

When the correlation between TGF*β*1 and each of the others parameters was examined in the overall population, no significant correlation could be observed between TGF*β*1 and the biological and clinicopathological variables, except the hormonal status. Thus, premenopausal patients were found to express higher TGF*β*1 levels than postmenopausal patients (114 *vs* 86 pg mg^−1^ protein, *P*=0.0006) (not shown). When patient population was subdivided according to pathological nodal status, TGF*β*1 remained correlated to the hormonal status in both node-negative (*P*=0.012) and node-positive (*P*=0.008) subgroups (Table 3). Moreover, a positive association between TGF*β*1 and PAi-1 (*P*=0.040) was observed in the node-negative population.

### Prognostic relevance

**Figure 1:**
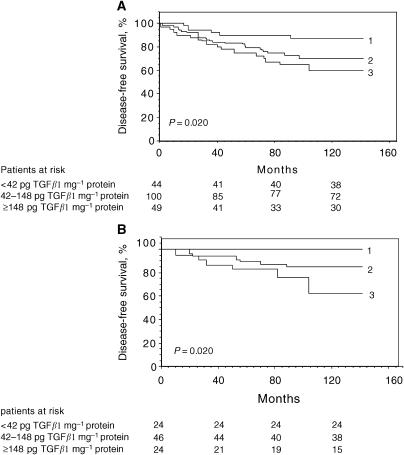
The probability of DFS for overall (**A**) and node-negative (**B**) populations according to TGF*β*1 levels. 1, negative (TGF*β*1<42 pg mg^−1^ protein); 2, low (42 pg mg^−1^ protein ⩽TGF*β*1 <148 pg mg^−1^ protein); 3, high (TGF*β*1⩾148 pg mg^−1^ protein).

The impact of TGF*β*1 on OS and DFS was determined in the overall population and node-negative/node-positive subsets. When 25th and 75th percentiles of the distribution were used as cutoff values, TGF*β*1 appeared significant (*P*=0.020) for DFS in the overall population (Figure 1A). The 10-year probability of DFS was 86.4% for patients with low TGF*β*1 levels (<42 pg mg^−1^ protein), 72% for the intermediate group (42–148 pg TGF*β*1/mg protein) and 61% for patients with TGF*β*1⩾148 pg mg^−1^ protein. The patients were then dichotomised according to their nodal status. While TGF*β*1 was found to have no significant impact on DFS in the node-positive subgroup (not shown), high TGF*β*1 levels were significantly associated with poor DFS in the node-negative population (*P*=0.02) (Figure 1B). Thus, among the node-negative patients with low TGF*β*1 level, no relapse (DFS=100%) were observed, whereas 17 and 38% relapses were observed for patients with intermediate and high TGF*β*1 expression levels, respectively. Unlikely, the level of TGF*β*1 had no impact on OS, neither in the overall population nor in the node-positive/node-negative groups (not shown).

A Cox multivariate analysis was performed to evaluate whether TGF*β*1 might significantly add to the contribution of the traditional prognostic factors. A significance level of 5% in the univariate analysis was chosen as the criterion for entering variables (SBR grade, nodal status, ER, uPA, PAi-1, TK and TGF*β*1) (not shown). The analysis was conducted in the overall population and node-negative/node-positive subgroups (Table 4). The analysis performed for OS revealed nodal status (*P*=0.0003) and SBR grade (*P*=0.0008) as independent parameters in the overall population. The prominent predictor for OS was SBR grade (*P*=0.004) and ER (*P*=0.020) in the node-positive population, and uPA (*P*=0.010) in the node-negative subgroup. In addition of nodal status (*P*=0.0002) and uPA (*P*=0.004), TGF*β*1 was independently associated to poor DFS in the overall population (*P*=0.005). Whereas PAi-1 appeared as the prominent independent predictor for the node-positive patients (*P*=0.019), the parameter associated with DFS in the population without node-infiltration was TGF*β*1 (*P*=0.010).

## DISCUSSION

The reduced response to TGF*β* in some tumour systems appears to involve multiple mechanisms, including loss of functional TGF-*β* receptor proteins (Grady *et al*, 1999; Fukai *et al*, 2003). In addition, mutations of downstream TGF-*β* signalling pathway genes have also been shown to result in a loss of responsiveness to TGF-*β*1 (Wang *et al*, 2000; Maliekal *et al*, 2003). In contrast to many other tumours, structural lesions of TGF*β* signal transducers appear to be rare in breast cancers (Chen *et al*, 1998; Xie *et al*, 2002; Jeruss *et al*, 2003). This suggests that, in a number of circumstances such as cell dedifferentiation, the normal function of TGF*β*1 in breast epithelial cells might be abrogated on behalf of oncogenic function.

Whereas TGF*β*1 seems to be confirmed as a marker of bad prognostic in a number of human tumours such as colorectal (Tsushima *et al*, 1996; Picon *et al*, 1998) and prostatic (Ivanovic *et al*, 1995; Shariat *et al*, 2004) cancers, the impact of TGF*β*1 on the progression of breast cancer remains uncertain. As for carcinomas in other organs, TGF*β*1 expression is often increased locally and systemically in advanced breast cancers, particularly at the leading invasive edge of the tumour and in metastasis (Dalal *et al*, 1993; Walker *et al*, 1994; Chakravarthy *et al*, 1999). Nevertheless, whereas the elevated expression of TGF*β*1 is described to associate with disease progression in a number of studies (Gorsch *et al*, 1992; Dalal *et al*, 1993; Walker *et al*, 1994), others studies failed to reveal diagnostic or predictive value of TGF*β*1 for breast cancer patients (Dublin *et al*, 1993; Murray *et al*, 1993; Kesari *et al*, 1999; Ghellal *et al*, 2000).

It is notable that, except when measured in plasma, most of the authors employed semiquantitative immunohistochemical staining to evaluate TGF*β*1 protein level in breast samples. In this study, we have measured for the first time the level of TGF*β*1 protein in breast tumour samples by ELISA, in order to examine potent correlations with clinical features. Using this assay, we found 94.3% of patients expressing TGF*β*1 with a range of 0–684 pg mg^−1^ protein and a median value of 86.7 pg mg^−1^ of protein.

In agreement with a number of previous studies, we show in the overall population that TGF*β*1 was correlated only with menopausal status. Thus, a moderate but significant increase of tumoral TGF*β*1 level was observed in premenopausal patients when compared to postmenopausal subgroup (*P*=0.0006). These data are in apparent opposition with some published studies, indicating that estradiol decreased the production of TGF*β*1 by breast cancer epithelial cells *in vitro* (Knabbe *et al*, 1987; Philips and McFadden, 2004). Nevertheless, the diminution of TGF*β*1 in postmenopausal patients might reflect adaptability process of tumoral cells to the profound hormonal modifications, which occur during menopause.

The increased expression of uPA has been reported to be associated with poor prognostic for patients with breast cancer (Duffy *et al*, 1998). Paradoxically, its inhibitor PAi-1 has also been described to contribute to the malignant phenotype of tumour cells (Look *et al*, 2002; Schrohl *et al*, 2004). Thus, PAi-1 might promote the development of tumoral angiogenesis through the stabilisation and maturation of new vessels (Bajou *et al*, 2004). Interestingly, TGF*β*1 was also found to be positively correlated to PAi-1 in the node-negative subgroup (*P*=0.040). The activity of PAi-1 is tightly regulated on the transcriptional level, and TGF*β*1 is the major regulator of PAi-1 expression and in turn of local PAi-1 activity (Westerhausen *et al*, 1991). Thus, in the node-negative population, the upregulation of PAi-1 by TGF*β*1 might constitute an early event that promotes further progression of breast tumours. This is in agreement with our data indicating that TGF*β*1 is an indicator of bad prognostic for breast cancer patients. Thus, multivariate analysis revealed that, after lymph node status (*P*=0.0002) and uPA (*P*=0.004), TGF*β*1 was an independent prognostic marker for DFS (*P*=0.005) in the overall population. Furthermore, TGF*β*1 remained the prominent prognostic factor in the node-negative population (*P*=0.010). In this late population, Kaplan–Meier curves further demonstrated that high level of TGF*β*1 was correlated with a shorter disease-free survival (*P*=0.020). Conversely, TGF*β* was not a prognostic factor for OS in the node-negative population. However, it has to be mentioned that at the cutoff date of the study, three deaths had been recorded in the node-negative subgroup. This is probably insufficient to distinguish a potential influence of TGF*β* on overall survival, in this population. Whereas clinical studies in breast cancers have led to conflicting results, our data suggest that TGF*β*1 has the potential to promote metastasis and recurrence for patients with breast carcinomas. It has to be noted that patients included in this study have not received modern chemotherapy, which could influence the outcomes. The fact that prognostic value of TGF*β*1 was observed in node-negative population strongly suggests that TGF*β*1 interferes at early stages of tumour progression, probably by making cell environment favorable for metastatic spread.

Although the lymph node status is one of the best prognostic factors in breast cancer, it is not sufficiently accurate to predict the clinical course of the disease. Indeed, 20–30% of node-negative breast cancer patients will experience disease recurrence and metastatic dissemination. Whereas numerous predictive factors have been characterised thus far, early prognostic markers that interfere at the beginning of tumour progression are scarce. The prognostic significance of high TGF*β*1 level on DFS observed in node-negative breast cancer patients suggest that the determination of tumoral TGF*β*1 status might help to identify a high-risk population early in tumour progression, for which a more appropriate therapy should be established. In this context, it appears fundamental to confirm the prognostic value of TGF*β* in a large cohort of node-negative patients. Furthermore, as total TGF*β* (active *plus* latent forms) has been measured in our study, it would be helpful to determine the respective role for latent and active TGF*β* as prognostic markers in breast cancers.

## Figures and Tables

**Table 1:** Patient characteristics

**Feature**	**Category**	**No. of patients**	**Percentage**
Total population		193	
Age (years)	⩽50	62	32.1
	>50	131	67.9
			
Hormonal status	Premenopausal	72	37.3
	Menopausal	121	62.7
			
Receptor status^a^	ER− PR−	21	10.9
	ER− PR+	7	3.6
	ER+ PR−	39	20.2
	ER+ PR+	126	65.3
			
Histology	Invasive duct	145	75.1
	Invasive lobular	48	24.9
			
Tumour size	T1	83	43.0
	T2	110	57.0
			
SBR grade	I	49	25.5
	II	102	52.8
	III	42	21.7
			
Nodal status	N−	94	48.7
	N+	57	29.6
	N++	42	21.7

a The subgroups ER+ include the patients with ER+ and ER++ (ER>15 fmol mg^−1^ protein), as defined in Materials and Methods section.

**Table 2:** Biological characteristics of the breast cancer population

**Variables**	**Range**	**Mean (IC)** a	**Q25**	**Q50**	**Q75**
*ER (fmol* *mg*^−*1*^ *protein*)					
Overall population	0–654	197 (23.9)	33	149	325
Premenopausal	1–455	122 (26.8)	22	87	205
Postmenopausal	0–654	218 (22.4)	67	254	377
					
PR (fmol mg^−1^ protein)	0–1000	156 (26.3)	8.7	67	274
TK (mU mg^−1^ protein)	4–2804	349 (66.9)	70	138	388
uPA (ng mg^−1^ protein)	0.01–1.39	0.28 (0.03)	0.11	0.20	0.38
PAi-1 (ng mg^−1^ protein)	0.27–54	8.73 (1.19)	3.7	6.0	10
TGF*β*1 (pg mg^−1^ protein)	0–684	121 (16.6)	42	86.7	148

a 95% confidence interval, CI.

**Table 3:** Relationship between TGF*β*1 and biological and clinicopathological variables

	**Node-negative patients**				
	***n***	**Range**	**Q_50_**	***P***	***ρ***
*Hormonal status*					
Premenopausal	35	8–567	132		
Postmenopausal	59	0–349	74	0.012	−0.259
					
*Tumour size*					
T1	61	0–349	74		
T2	33	3–367	91	0.725	0.036
					
*SBR grade*					
I	30	3–349	100		
II	48	0–367	82		
III	16	3–337	79	0.885	0.015
					
*ER*					
Negative	17	17–259	75		
Low	56	0–367	85		
High	21	9–349	117	0.906	0.094
					
*PgR*					
Negative	30	3–341	72		
Positive	64	0–349	104	0.696	0.041
					
*TK*					
Negative	26	3–349	102		
Low	50	0–337	69		
High	18	15–684	132	0.293	0.108
					
*uPA*					
Negative	28	0–303	72		
Low	42	7–349	107		
High	24	4–337	79	0.380	0.091
					
*PAI-1*					
Negative	26	0–167	72		
Now	47	3–366	81		
High	21	3–684	149	0.040	0.211
					

	**Node-positive patients**				
	***n***	**Range**	**Q_50_**	***P***	***ρ***
*Hormonal status*					
Premenopausal	37	13–684	120		
Postmenopausal	62	2–405	71	0.008	−0.265
					
*Tumour size*					
T1	22	5–679	88		
T2	77	2–684	76	0.943	−0.007
					
*SBR grade*					
I	19	4–346	76		
II	54	2–643	98		
III	26	2–684	62	0.680	−0.041
					
*ER*					
Negative	10	14–433	107		
Low	62	3–643	83		
High	27	3–684	89	0.728	0.035
					
*PgR*					
Negative	32	3–684	101		
Positive	67	2–678	87	0.929	0.009
					
*TK*					
Negative	20	3–257	83		
Low	42	2–387	107		
High	37	10–325	82	0.749	0.032
					
*uPA*					
Negative	17	3–348	87		
Low	56	2–684	96		
High	26	6–151	90	0.793	−0.027
					
*PAI-1*					
Negative	21	3–643	82		
Now	50	4–341	89		
High	28	6–676	100	0.977	−0.003

*n*=number of patients; Q_50_, median values.

**Table 4:** Cox multivariate analysis of OS and DFS, in overall population (*n*=193), and in node-positive (*n*=99) and node-negative patients (*n*=94)

		**DFS**		**OS**	
**Variable category**	**Coding**	**HR (CI)**	***P***	**HR (CI)**	***P***
**Overall population**					
*Nodal status*					
N−	0	1.00		1.00	
N+	1	1.84 (1.33–2.54)		2.31 (1.46–3.66)	
N++	2	3.39 (1.77–6.45)	0.0002	5.34 (2.13–13.4)	0.0003
					
*SBR grade*					
I	0			1.00	
II	1			2.89 (1.55–5.37)	
III	2			8.35 (2.42–28.8)	0.0008
					
*UPA*					
Negative	0				
Low	1	2.00 (1.25–3.21)			
High	2	4.02 (1.57–10.3)	0.004		
					
*TGFβ1*					
Negative	0	1.00			
Low	1	1.83 (1.19–2.81)			
High	2	3.36 (1.43–7.91)	0.005		
					
**Node-positive patients**					
*PAi-1*					
Negative	0	1.00			
Low	1	2.09 (1.26–3.46)			
High	2	4.36 (1.58–11.7)	0.019		
					
*SBR grade*					
I	0			1.00	
II	1			3.07 (1.43–6.62)	
III	2			9.46 (2.47–45.2)	0.004
					
*ER*					
Negative	0			1.00	
Low	1			0.45 (0.22–0.9)	
High	2			0.20 (0.05–0.81)	0.020
					
**Node-negative patients**					
*UPA*					
Negative	0			1.00	
Low	1			3.75 (0.83–16.9)	
High	2			14.1 (0.69–285.5)	0.010
					
*TGFβ1*					
Negative	0	1.00			
Low	1	2.90 (1.19–7.10)			
High	2	8.41 (1.41–50.4)	0.010		

Candidate variables in the Cox model are listed in Results. HR, hazard ratio; CI, 95% confidence interval.
